# Removal of a Large Fibro-Cystic Tumor of the Uterus by Gastrotomy

**Published:** 1872-06

**Authors:** T. Gaillard Thomas

**Affiliations:** Professor of Obsterics and Diseases of Women and Children in the College of Physicians and Surgeons, New York


### Removal of a Large Fibro-Cystic Tumor of the Uterus by Gastrotomy.

By T. Gaillard Thomas, M. D., Professor of Obsterics and Diseases of Women and Children in the College of Physicians and Surgeons, New York. Recorded by Dr. Matthew D. Mann, House Physician.

"E. K., aged twenty-eight, married, living in New Jersey, admitted to the Strangers' Hospital September 24, 1871. Patient has been married ten years, but never been pregnant. Her menses appeared at the usual time, and have always recurred regularly.

A year ago she noticed some swelling in the lower part of her abdomen, originating in the left side. This increased quite slowly during the first six months, and attracted very little attention. Since last spring the growth developed rapidly until its present size. Within a short time she has complained of dyspnœa or orthopnœa, of considerable pain in the back, and of marked debility.

On admission, patient weak but in good spirits; pulse over 100. The abdomen is distended but very tense; the umbilical depression not effaced. There is absence of resonance all over the front of the abdomen up to a point midway between the umbilicus and the ensiforn cartilage, and distinct fluctuation. The tumor resembles in shape and size the uterus in the eighth month of pregnancy, and is supposed to be an ovarian cyst requiring immediate measures for relief, on account of the extreme tension and the pain which this induces.

*Operation.*—On the 25th of September, the patient having been etherized, the tumor was removed by Dr. Thomas, in the presence of Drs. Peaslee, Sands, Draper, Markoe, Brown, and a large number of students. An incision of three inches was made in the median line midway between the umbilicus and pelvis down to the sac. A sound was then swept around the tumor, which demonstrated the fact that it was free of attachments. The tumor was then tapped, and there flowed away about four quarts of a chocolate colored fluid, which was found to consist of bloody serum. The wall of the supposed ovarian cyst was found to be so very thick that, after removal of the fluid, difficulty was experienced in withdrawing the sac through the opening. This was, therefore, prolonged to five or six inches and the sac drawn out. To the surprise of the operator, it was found to be a sac with very thick walls growing from the fundus uteri, and disconnected with the ovaries, both of which were healthy. The only explanation which could be given of its developement, was that a tumor originally solid had undergone cystic degeneration or liquefaction in its centre, and thus taken on a cystic character. Hæmorrhage had unquestionably occurred within the sac thus formed, for a large amount of blood was found to exist in the fluid removed by tapping. The walls of this cyst was estimated as about half an inch thick.

The removal of the tumor presented great difficulties, for no pedicle existed. This was accomplished by that species of enucleation recommended by Dr. Miner, of Buffalo, N. Y. An incision having been made through the outer envelope of the tumor, the operator stripped this back on both sides, and by this means enucleated the tumor. From the walls of the enveloping sac thus left, a good deal of hæmorrhage occurred, but this was controlled by one or two ligatures, free application of solution of persulphate of iron and exposure to the atmosphere. The walls of this envelope of the tumor were then ligated by silk and returned to the abdomen. The abdominal wound was closed by silver sutures, except at the lower extremity, into which a small tent was inserted to facilitate drainage and washing out of the peritoneum, which the operator thought would almest assuredly be demanded.

8 P. M.—Patient recovers slowly from the ether; pulse 108; is kept quiet with morphine."

"Three months after the operation she presented herself at the hospital, looking fat and healthy. So changed was she in appearance that it was difficult ar first to recognize her."

